# Caregiver Choice and Caregiver Outcomes: A Longitudinal Study of Irish Spousal Dementia Caregivers

**DOI:** 10.3389/fpsyg.2019.01801

**Published:** 2019-08-13

**Authors:** Maria M. Pertl, Aditi Sooknarine-Rajpatty, Sabina Brennan, Ian H. Robertson, Brain A. Lawlor

**Affiliations:** ^1^Royal College of Surgeons in Ireland, Dublin, Ireland; ^2^Trinity College Institute of Neuroscience, Trinity College Dublin, Dublin, Ireland

**Keywords:** dementia, caregiving – informal, institutionalization, psychological wellbeing, benefit finding, choice, care, older adult

## Abstract

**Background:**

The perception of choice in becoming a caregiver may impact on caregiver psychological and physical health. We determined the proportion of spousal dementia caregivers who felt they had a choice, and examined whether lack of choice in taking up the caregiving role and the perceived degree of choice in caregiving predicted caregiver health and wellbeing and care-recipient placement in long-term care at 1-year follow-up.

**Methods:**

We performed secondary analyses of data from DeStress, a longitudinal study of 251 spousal dementia caregivers in Ireland. We used multivariate logistic and linear regression analyses to examine whether lack of choice (a dichotomous item) and/or the perceived degree of choice (a 9-point scale) at baseline predicted caregiver health (number of chronic health conditions; self-reported health) and wellbeing (e.g., burden, anxiety, depression, stress, and positive aspects of caregiving) and care status (continued care at home or placement in long-term care) at follow-up.

**Results:**

The vast majority of caregivers (82%) reported that they had no choice in taking up the caregiving role. Nevertheless, nearly three-quarters (74%) responded above the midpoint on the rating scale (Mean = 6.82, *SD* = 3.22; Median = 9; Mode = 9), indicating they provided care voluntarily. Caregivers who reported a greater degree of choice were more likely to still be providing care at home at follow-up and to identify benefits from providing care. Neither choice nor degree of choice predicted any other caregiver outcomes.

**Conclusion:**

For the vast majority of spousal dementia caregivers, taking up the caregiving role is not perceived as a choice; yet, most report performing this role voluntarily. Thus, facilitating greater choice may not necessarily diminish the key contribution family caregivers make to the care system. Although we found no evidence that caregiver choice predicted more positive caregiver health and wellbeing, the perception of choice is important in and of itself, and may benefit caregivers by facilitating the identification of positive aspects of care and be a factor in delaying care-recipient placement in long-term care. Future research should be especially mindful of how caregiver choice is assessed and how this may affect the resulting prevalence of choice.

## Introduction

Unpaid family members provide the vast majority of care for the increasing numbers of people living with dementia worldwide (Roth et al., 2005; World Health Organization, and Alzheimer’s Disease International, 2012). In Ireland, 63% of the estimated 55,000 people with dementia (Pierse et al., 2018) live in the community where they are cared for by family members or friends (Cahill et al., 2012). While stated Government policy is to support care in the community for older people, including those living with dementia (Department of Health, 2014b); in reality, home care in Ireland is a family care system that is merely supplemented by the state. Of the €1.65bn per year in estimated costs of dementia care, nearly half (48%) can be attributed to the opportunity costs of family caregivers, whilst 43% is attributable to residential care (Connolly et al., 2014). In 2010, family and friends provided an estimated 81 million hours of care for people with dementia, saving the Irish government €807 million (Connolly et al., 2014). The reliance on family caregivers and their contribution as the main providers of dementia care can therefore not be overstated.

The Irish National Dementia Strategy acknowledges the excessive demands placed on family dementia caregivers and recognizes the need to safeguard their own health, psychological wellbeing, and social life (Department of Health, 2014a). Indeed, it is widely documented that providing dementia care can be extremely demanding and stressful for caregivers, and that this may negatively impact on caregivers’ own physical health and emotional well-being (Pinquart and Sorensen, 2003; Berglund et al., 2015). In this context, the perception of choice in taking on the responsibility of care may play an important role (Weinstein and Ryan, 2010). Feelings of agency, autonomy, and freedom of choice are consistent predictors of well-being, life-satisfaction and happiness (Welzel and Inglehart, 2010; Fischer and Boer, 2011). However, few studies have examined relationships between choice, or the lack of it, and caregiver health and well-being. Furthermore, it is not clear what proportion of family caregivers actually provide dementia care on voluntary basis.

We define caregiver choice as the extent to which an individual believes they had the freedom to choose to take up the responsibility of care (Al-Janabi et al., 2018). While it is generally recognized that caregivers should have a choice in whether to provide care or not, as well as the extent and nature of the care they provide; in reality, given the increasing demand for dementia care and the already heavy reliance on unpaid family members and friends as the main providers of care, this is unlikely to be the case for many caregivers. Caregivers are often implicitly treated as free resources; they are valued, but largely because without them the current care system is completely unsustainable. While policy developments recognize the importance of supporting caregiver wellbeing, this is largely in the context of ensuring the continuation of care and the well-being, and interests of the care-recipient (Arksey and Glendinning, 2007). Indeed, despite increasing emphasis on the importance of respecting the choices for care-recipients and responding to their individual needs, choice for caregivers has been largely overlooked. For example, a key objective set out by the Irish National Dementia Strategy (Department of Health, 2014a) is that “people with dementia should be facilitated and supported to live and die well in their chosen environment including their own home or nursing home if that is their choice” (p. 25). However, there is no equivalent acknowledgement that caregivers should also be able to exercise personal choice in terms of whether and how they provide care. Caregiver choice is constrained by a multitude of factors that include, but are not limited to, caregivers’ personal values and/or societal norms of responsibility and reciprocity, which may lead to a sense of duty to provide care; care-recipients’ own wishes for their care; the lack of tangible assistance from other family members and/or the state; and financial constraints and/or the inadequacy of available services that limit alternative care options (Robinson-Whelen and Kiecolt-Glaser, 1997; Al-Janabi et al., 2018; Rand et al., 2019). Furthermore, opportunities for choice may vary depending on the nature of the care-recipients’ condition and the level of care that is required.

A large United States study on a mixed sample of informal caregivers found that over half of caregivers (54%) reported they had a choice in taking up the caregiver role (Winter et al., 2010). A similar prevalence of caregiver choice (56%) was found in another US study on caregivers of older adults specifically (Schulz et al., 2012). These contrast with a more recent United Kingdom study (Al-Janabi et al., 2018) in which more than 80% of survey respondents felt they had a choice to provide care; though only a third of respondents felt they had a free choice that was not constrained by a sense of duty, the unavailability of other caregivers, or financial factors. Such research suggests that a substantial number of caregivers do feel they have a choice in providing care. Nevertheless, none of these studies specifically focused on dementia caregivers; all included care-recipients with a variety of conditions, caregivers who were not necessarily the main providers of care, and a mix of caregiver/care-recipient relationships including, for example, adult children caring for parents and non-relative caregivers. Furthermore, in the two studies that reported data on the level of care, the vast majority of caregivers provided less than 20 h of care per week (Winter et al., 2010; Al-Janabi et al., 2018). Given the unique challenges associated with dementia care and the finding that dementia caregivers, and spousal caregivers in particular, provide a higher level of care, have less leisure time, report greater withdrawal of support from family and friends and less affectionate social support, more interrupted sleep, and more depressive symptoms than caregivers of people without dementia (Nordtug et al., 2013; Moon and Dilworth-Anderson, 2015), it is likely that the prevalence of caregiver choice is lower among this population. However, to our knowledge, no previous studies have reported on the prevalence of choice among dementia caregivers specifically, and little is known about the nature of the relationship between dementia caregiver choice and caregiver health and wellbeing outcomes.

Previous studies on broader caregiver samples suggest that the absence of caregiver choice is a significant predictor of poorer caregiver outcomes on measures including life-satisfaction, happiness, quality of life, and capability (Al-Janabi et al., 2018); emotional stress, health impact and physical strain (Schulz et al., 2012); and burden and stress (Winter et al., 2010). Furthermore, studies that have examined reasons for caregiving, which are tied to caregiver choice, have also found associations with poorer caregiver wellbeing. For example, the perceived lack of availability or suitability of other care options predicted poorer care-related quality of life in a mixed sample of English caregivers, though no significant relationships were observed between personal choice/willingness to care, and measures of quality of life or strain (Rand et al., 2019). Other studies suggest that intrinsic motivations to care are associated with positive caregiver mental health, while external motivations or pressures predict negative outcomes such as stress, anxiety, depression, and anger (Lyonette and Yardley, 2003; Losada et al., 2010; Romero-Moreno et al., 2011; Kim et al., 2015).

To our knowledge, only one previous study has explicitly examined the role of choice among spousal dementia caregivers specifically; Robinson-Whelen and Kiecolt-Glaser (1997) found that a lower perception of the degree of choice (assessed on a 9-point scale) predicted greater caregiver stress, as well as greater depression among caregiving husbands. Such research suggests that the ability to exercise choice is important not only in and of itself, but also because it may impact on caregiver outcomes. However, while Robinson-Whelen et al. collected follow-up measures of caregiver distress, their ability to examine relationships with baseline choice was limited by attrition and missing outcome data. Of the limited previous research outlined above that examined relationships between choice or reasons for caregiving and caregiver outcomes, only one study (Kim et al., 2015) was based on longitudinal data; hence it is possible that wellbeing factors that were assessed were driving the perception of choice/reasons for care rather than vice versa or that caregivers with better well-being were more likely to have the capacity for choice. Prospective studies on relationships between choice and caregiver outcomes are therefore needed.

In addition to impacting on caregiver health and wellbeing – or perhaps through these factors -, perceptions of caregiver choice may also play a role in the premature cessation of care in the community. Indeed, previous research has indicated that caregiver “role captivity” – fulfilling a role because of an obligation rather than by choice – predicts nursing home placement among persons with Alzheimer’s disease (Aneshensel et al., 1993). However, no previous research has explicitly examined relationships between the perception of choice and cessation of dementia care in the community.

Given that the prevalence of caregiver choice among dementia caregivers is not known, our aim was to determine the proportion of caregivers who felt they had a choice in taking up the responsibility of care for their spouse with dementia. Furthermore, building on previous cross-sectional research, we sought to examine whether the perception of choice in taking up the caregiving role and the degree of choice predicted a variety of caregiver health and wellbeing outcomes, as well as care-recipient placement in long-term care, at 1-year follow-up. We hypothesized that lack of choice and a lower degree of choice would predict poorer caregiver outcomes.

## Materials and Methods

### Participants and Procedure

Participants were recruited for a longitudinal study (the DeStress study) on the relationship between caregiver stress and cognitive functioning (Pertl et al., 2017; see O’Sullivan et al., 2018). Caregivers over the age of 50, who were providing care at home for a spouse or common-law partner with a formal diagnosis of dementia of the Alzheimer’s type, Parkinson’s disease, or other primary degenerative dementia, were eligible to participate.

Participants were recruited from all over Ireland through a broad range of channels, including media advertisements, as well as community gatekeepers and organizations for caregivers, dementia and/or older people. Of the 370 persons identified who were eligible, 252 (68%) participated. All data, unless otherwise specified, were collected at two study time-points (baseline and 1-year follow-up) using a combination of a telephone health survey, a postal questionnaire, and a face-to-face assessment.

The study protocol was approved by the Trinity College Dublin School of Psychology Ethics Committee. Caregivers’ participation in the study was completely voluntary and participants were free to withdraw from the study at any time. Participants were also free to decline to provide some or all information requested as part of the study. All participants gave written informed consent.

### Measures

#### Participant Demographics and Care-Recipient Factors

Sociodemographic factors including age, sex, and level of education (years) were recorded at baseline. Items from the *RUD4* (Wimo et al., 2013) were used to assess length of care time (in months) and the average number of caregiving hours per day. Caregiving status (still caring at home, spouse in long-term care, or bereaved) was recorded when participants were contacted for follow-up assessment.

The severity of the care-recipients’ behavioral and psychological symptoms associated with dementia (BPSDs) was assessed using the *neuropsychiatric inventory questionnaire* (NPI-Q) (Kaufer et al., 2000). The NPI-Q covers 12 BPSDs (e.g., agitation/aggression, apathy/indifference, disinhibition, and night-time behavioral disturbances) the severity of which are rated from 1 (mild) to 3 (severe) if a symptom is present, or scored as 0 if not present. The NPI-Q has demonstrated adequate test-retest reliability and convergent validity (Kaufer et al., 2000).

The *Bristol Activities of Daily Living Scale* (BADLS) (Bucks et al., 1996) was used as a measure of care-recipient functional impairment. The 20-item questionnaire was developed specifically as a brief, self-rated activities of daily living (ADL) scale for caregivers of persons with dementia and includes both ADLs (e.g., eating and dressing) and instrumental ADLs (IADLs; e.g., managing finances). Each of the 20 items is rated on four responses that refer to different levels of ability, resulting in a minimum total score of 0 (totally independent), and a maximum score of 60 (totally dependent). The BALDS has demonstrated good test-retest reliability and content validity (Bucks et al., 1996), as well as sensitivity to change, and expected relationships with measures of cognition (Byrne et al., 2000).

#### Caregiver Choice

Perceived choice in caregiving was assessed with a single yes/no item (“Do you feel you had a choice in taking on the responsibility of caring for your spouse”?), which has been used in previous research (Winter et al., 2010; Schulz et al., 2012). An additional item was also included to assess the degree of choice (“How voluntary do you consider your caregiving to be?”) measured on a 9–point rating scale from 1 (“no choice”) to 9 (“completely voluntary”) (Robinson-Whelen and Kiecolt-Glaser, 1997). Only participants who were still providing care at home 1 year later completed these measures again at follow-up.

#### Caregiver Physical Health

A profile of participants’ health was obtained using items from the cognitive function and aging study (Yip et al., 2006) and the *Christensen health screening questionnaire* (Christensen et al., 1992); the total number of chronic health conditions participants had was recorded. In addition, self-reported health was assessed using a 5-point Likert Scale, rated from excellent to poor (Romero-Ortuno et al., 2010).

#### Caregiver Psychological Wellbeing

*Caregiver burden* was measured using the *Zarit burden interview* (Zarit et al., 1980), a 22-item questionnaire that produces scores ranging between 0 (no burden) to 88 (severe burden).

*Stress* was measured using the 4-item *perceived stress scale* (PSS-4) (Cohen et al., 1983), a widely used psychological instrument that assesses how unpredictable, uncontrollable, and overloaded participants find their lives. The total score ranges from 0 – 16, with higher scores indicating more perceived stress. The PSS-4 has good internal reliability and adequate test-retest reliability, and is suggested for use in cases where very short scales are required (Cohen et al., 1983).

*Anxiety* was assessed using the 7 anxiety items on the *Hospital Anxiety and Depression Scale* (HADS-A) (Zigmond and Snaith, 1983). The HADS-A items are scored on 4-point (0 - 3) response scale with a maximum score of 21. Higher scores indicate more severe anxiety. Investigations of the factor structure, discriminant validity, and internal consistency have shown that the psychometric properties of the HADS are excellent (Mykletun et al., 2001; Bjelland et al., 2002).

*Depression* was assessed using the *Centre for Epidemiological Studies Depression* (CES-D) scale (Radloff, 1977). The CES-D consists of 20 items assessing depressed affect, lack of positive affect, somatic symptoms, and interpersonal difficulties during the preceding week. A total summed score (ranging from 0 to 60) can be calculated with higher scores indicating greater depressive symptomology. The CES-D is internally consistent, moderately stable over time, and strongly correlated with other measures of depression (Roberts and Vernon, 1983; Lewinsohn et al., 1997).

*Self-efficacy* for symptom management was assessed using the *Fortinsky dementia-specific caregiver self-efficacy scale* (Fortinsky et al., 2002). Scores on six items, assessed on 10-point Likert scales, are summed with higher scores indicating greater self-efficacy. The measure has demonstrated good internal consistency (Fortinsky et al., 2002).

*The Positive Aspects of Caregiving* scale (Tarlow et al., 2004) was used to assess caregivers’ perception of benefits associated with their caregiving, such as feeling useful, feeling appreciated, and finding meaning. The scale consists of 9 items rated on a 5-point Likert scale; a total score (from 9 to 45) is obtained by summing scores, with higher scores indicating more positive caregiving appraisals. The scale has demonstrated high internal reliability (Tarlow et al., 2004).

*Quality of Life* was calculated using the *CASP-12*, a short version of the CASP-19, (Wiggins et al., 2008), which focuses on three aspects of life: control and autonomy, realization, and pleasure. The 12-items were rated on a 4-point Likert scale, producing a total score between 12 and 48, with 48 indicating better quality of life.

### Analysis

Descriptive statistics were calculated for caregiver choice and degree of choice as well as for caregiver and care-recipient characteristics (sex, age, education, duration of and time per day spent caregiving, care-recipient functional impairment, and BPSD severity). The relationships between caregiver and care-recipient characteristics and the caregiver choice measures were examined using *t*-tests, Chi-square analysis and Pearson correlations.

Multivariate logistic regression analysis was carried out to examine whether caregiver choice and/or the perceived degree of choice at baseline predicted placement in long-term care at follow-up amongst those who were not bereaved in the intervening period, and whose care status was known. Model fit was evaluated with the Hosmer-Lemeshow test and compared with standard χ^2^ likelihood ratio statistics. Caregiver age, sex and education; the duration of caregiving; and care-recipient level of functional impairment and BPSD severity were included as covariates.

Relationships between the hypothesized predictors (caregiver choice and degree of choice) and caregiver health and well-being variables (number of comorbidities, self-rated health, burden, stress, anxiety, depression, self-efficacy, positive aspects of caregiving, and quality of life) were examined at baseline and at follow-up using Pearson correlations amongst those still providing care at home. In order to minimize potential confounds, only those still providing care at follow-up were included in this analysis; however, results were equivalent when those who were bereaved and those whose spouse was in long-term care at follow-up were included. Multivariate linear regression analysis was conducted to test whether baseline lack of choice and/or perceived degree of choice predicted caregiver health and wellbeing outcomes at follow-up. In addition to the covariates included in the logistic regression models, baseline measures of the relevant health, and well-being outcome was controlled in each model.

## Results

### Caregiver and Care-Recipient Characteristics

The participant socio-demographic characteristics and the care-recipient characteristics are presented in Table 1. Approximately two thirds of participants were female and the mean age of the sample was just under 70 years of age. On average, participants had been providing care for approximately 5 years and were spending 12 h per day on caregiving activities.

**TABLE 1:** Caregiver and care-recipient characteristics at baseline.

**Caregiver and care-recipient characteristics**	**Number (%)/mean (SD)**
Sex (female)	163 (64.7%)
Age	69.65 (7.86)
Education (years)	13.27 (3.68)
Some primary not complete	15 (6%)
Primary or equivalent	25 (9.9%)
Inter/junior certificate (some HS)	49 (19.4%)
Leaving certificate (HS diploma)	46 (18.3%)
Diploma or certificate	54 (21.4%)
Degree	36 (14.3%)
Postgraduate/higher degree	27 (10.7%)
Duration of caregiving (months)	59.44 (39.29)
Hours of caregiving per day	12.09 (6.12)
Severity of care-recipient BPSDs	11.56 (7.08)
Functional impairment (ADL/IADL)	27.49 (13.39)
**Caregiver choice**	
Perceived choice in providing care (“yes” respondents)	45 (17.9%)
Perceived degree of choice (scale 1 – 9)	6.89 (3.17)
Response above the midpoint (i.e., care perceived to be voluntary)	182 (72.2%)

*Higher scores indicate greater severity of BPSDs and functional impairments; ADL, activities of daily living; IADL, instrumental activities of daily living; BPSDs, behavioral and psychological symptoms associated with dementia.*

### Prevalence of Caregiver Choice

**FIGURE 1:**
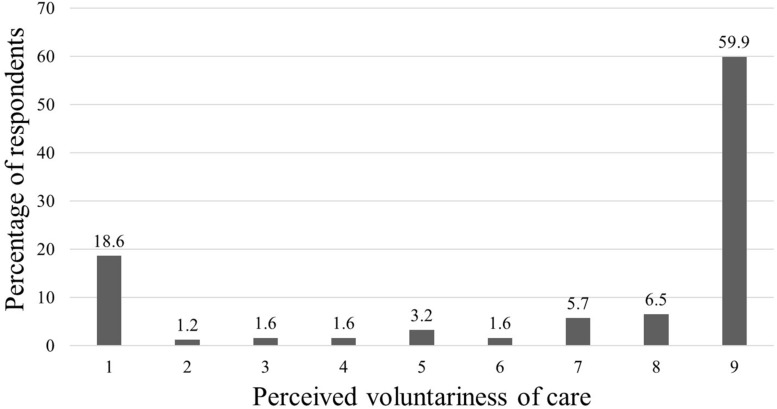
Distribution of responses on the perceived voluntariness of care scale at baseline.

Less than a fifth of caregivers (18%) reported that they had a choice in taking up the caregiving role (see Table 1). Nevertheless, nearly three-quarters of caregivers responded above the midpoint on the degree of choice scale with an overall mean of 6.82 (*SD* = 3.22; Median = 9; Mode = 9; see Figure 1). Neither the prevalence of choice, nor the perceived degree of choice, differed by sex, age, education, duration of caregiving, or care-recipient functional impairment and BPSD severity, *p* > 0.05 (results not presented).

Among those who were still providing care at home 1 year later, 23.3% agreed at follow-up that they had a choice in taking up the caregiving role. While 81.2% (*n* = 121) gave the same response to this item at baseline and follow-up, 7.4% (*n* = 11) of caregivers reported at follow-up that they had no choice when they had previously reported choice and 11.4% (*n* = 17) reported choice at follow-up when they had previously reported no choice. The perceived degree of caregiver choice did not change significantly from baseline to follow-up (Mean difference = 0.22, *SD* = 3.26), *t*(145) = 0.81, *p* < 0.05.

### Caregiver Choice and Degree of Choice as Predictors of Placement in LTC at Follow-Up

At follow-up 177 participants were still providing care for their spouse at home and 41 had moved their spouse into LTC, giving a combined sample of 218 participants. Bivariate correlations indicated that neither caregiver choice nor perceived degree of choice at baseline were significantly associated with placement in LTC at follow-up (*r* = −0.07 and −0.12, respectively, *p* > 0.05).

Caregiver choice at baseline was not a significant predictor of placement in LTC in a logistic regression model that adjusted for covariates (results not presented); however, the perceived degree of choice was (see Table 2). Specifically, a one-unit increase in perceived degree of choice at baseline was associated with a 12% lower likelihood of the care-recipient being in LTC 1 year later. The model was a good fit to the data, as indicated by a non-significant χ^2^ value for the Hosmer-Lemeshow test; however, while the inclusion of degree of choice in Block 2 made a statistically significant contribution to the model (χ^2^ = 4.52, *p* < 0.05), the model explained little additional variance and the percentage of cases correctly classified did not increase substantially over a model that included only the demographic covariates.

**TABLE 2:** Logistic regression of degree of caregiver choice at baseline and placement in LTC at follow-up.

	**Block 1**			**Block 2**		
		
	***B***	**SE**	**OR**	***B***	**SE**	**OR**
**Predictor variables**						
Constant	−9.15	2.51	0.001	−9.33	2.57	0.001
**Covariates**						
Sex	0.49	0.44	1.64	0.52	0.44	1.69
Age	0.07	0.03	1.07^*^	0.01	0.03	1.08^∗∗^
Education	0.01	0.06	1.00	0.01	0.06	1.01
Duration of caregiving	−0.01	0.01	0.99	−0.01	0.01	0.99^*^
Impairment on ADL/IADLs	0.09	0.02	1.09^∗∗∗^	0.09	0.02	1.10^∗∗∗^
BPSD severity	0.05	0.03	1.05	0.05	0.03	1.05
**Degree of choice**				−0.13	0.06	0.88^*^
−2LL	168.06 χ^2^ = 32.27, df = 6, *p* = 0.001			163.55 χ^2^ = 36.79, df = 7, *p* = 0.001		
Nagelkerke *R*^2^	23.3%			26.3%		
Hosmer-Lemeshow test	*p* = 0.12			*p* = 0.59		
Classification accuracy	81.2%			82.6%		

ADL, activities of daily living; IADL, instrumental activities of daily living; BPSDs, behavioral and psychological symptoms associated with dementia; ^*^p < 0.05, ^∗∗^*p* < 0.01, ^∗∗∗^*p* < 0.001.

### Caregiver Choice and Degree of Choice as Predictors of Caregiver Health and Wellbeing at Follow-Up

Of the 177 caregivers who were still providing care at home at follow-up, 151 (85%) participated in the follow-up assessment. Bivariate correlations between baseline caregiver choice and degree of choice and follow-up caregiver health and wellbeing are presented in Table 3. Caregiver choice was not significantly associated with any health and wellbeing variables, while degree of choice was only significantly associated with the perception of positive aspects of care, caregiver burden, and self-efficacy. The same pattern of results was noted among male and female caregivers (results not presented).

**TABLE 3:** Descriptive statistics for caregiver health and wellbeing at follow-up and Pearson correlations with caregiver choice and degree of choice at baseline (*n* = 151).

**Caregiver health and wellbeing**	**No. (%)/mean (SD)**	***r***	
		
		**Choice**	**Degree of choice**
No. of chronic health conditions	2.75 (1.77)	−0.01	0.11
Self-rated health	2.88 (0.95)	−0.01	–0.08
Caregiver burden	38.68 (15.74)	−0.16	–0.30^∗∗^
Stress	5.87 (3.26)	−0.01	–0.11
Anxiety	6.79 (4.35)	0.04	–0.05
Depression	15.40 (10.72)	0.03	–0.08
Self-efficacy	33.95 (12.81)	0.08	0.33^∗∗∗^
Positive aspects of caregiving	29.71 (9.33)	0.10	0.33^∗∗∗^
Quality of life	34.59 (6.97)	−0.02	0.08

Higher scores indicate greater self-efficacy; more severe burden, stress, anxiety, and depression; more positive aspects associated with caregiving and better quality of life. ^*^^*^p < 0.01, ^∗∗^^*^p < 0.001.

In multivariate models, caregiver choice did not significantly predict any caregiver health and well-being outcomes (results not presented); while the perceived degree of choice only significantly predicted the perception of positive aspects of care. Specifically, caregivers who reported greater voluntariness in taking up the caregiving role were more likely to identify benefits as a result of providing care at follow-up (see Table 4). Baseline age, sex, education, duration of caregiving, care-recipient functional impairment and BPSD severity explained 12% of the variance in positive aspects of care at follow-up, *F*(6,138) = 4.34, *p* < 0.001. The addition of baseline positive aspects of caregiving in step 2 explained a further 34% of the variance, *F*(7,137) = 19.71, *p* < 0.001. Finally, the addition of degree of choice in step 3 increased the explained variance by 2%, *F*(8,136) = 18.45, *p* < 0.001.

**TABLE 4:** Hierarchical multiple regression analyses of Positive Aspects of Caregiving at follow-up.

**Predictors (all at baseline)**	**Step 1 β**	**Step 2 β**	**Step 3 β**
**Covariates**			
Sex	−0.21^*^	−0.08	−0.10
Age	0.15	0.01	0.01
Education	−0.06	−0.04	−0.04
Hours of caregiving per day	−0.03	−0.01	−0.01
Functional impairment ADL/IADL	0.15	0.08	0.07
Severity of care-recipient BPSDs	−0.15	−0.07	−0.06
Positive aspects of caregiving		0.64^∗∗∗^	0.633^∗∗∗^
Degree of choice			0.15^*^
	*R*^2^ = 0.12^∗∗∗^	*R*^2^ = 0.48^∗∗∗^	*R*^2^ = 0.49^*^

ADL, activities of daily living; IADL, instrumental activities of daily living; BPSDs, behavioral and psychological symptoms associated with dementia; ^*^p < 0.05, ^∗∗^^*^p < 0.001.

## Discussion

Our findings indicate that the vast majority of caregivers – less than a fifth – felt that they had a choice in taking up the responsibility of care for their spouse with dementia. This was consistent at follow-up among those still providing care at home. The high prevalence of perceived lack of choice is striking, yet perhaps not totally unsurprising given the heavy reliance on family caregivers as the main providers of dementia care in the social care system in Ireland, and the limited availability of dementia services and supports that are a prerequisite for choice.

The substantially lower prevalence of caregiver choice we observed compared to previous studies (Winter et al., 2010; Schulz et al., 2012; Al-Janabi et al., 2018) is likely to reflect the nature of our sample. While all participants in the current study were the primary caregiver for a person living with dementia, previous studies included caregivers who were not necessarily the main care providers and, furthermore, provided care for a more diverse range of care-recipients who needed, on average, fewer hours of care per day. The perception of caregiver choice is likely to be more curtailed when the care-recipient has a more debilitating condition and greater care needs (Schulz et al., 2012; Al-Janabi et al., 2018). Nevertheless, the extent of care-recipients’ functional impairment and BPSD severity were not significantly associated with the perception of choice in our study.

Previous studies also included non-relative caregivers, while our sample consisted exclusively of spousal caregivers whose perceived freedom to choose is likely to be more constrained by a sense of reciprocity and responsibility than other types of caregivers. This is supported by previous findings that spousal and parental relationships with a care-recipient were significantly associated with a lack of choice (Schulz et al., 2012). Furthermore, our participants were all co-habiting, which is likely to restrict freedom of choice further still. These differences in study populations, and the relative homogeneity of our sample compared to other studies, may also explain why we did not observe relationships between caregiver choice and sex, age, or care duration.

Caregiver choice is important; yet, enabling unpaid caregivers to exercise free choice with regards to caregiving presents a significant problem if it leads to decreases in unpaid caregiving activities (Arksey and Glendinning, 2007). The provision of alternative care options is itself constrained by the cost of dementia care in the context of limited resources. Nevertheless, our findings suggest that facilitating greater freedom of choice would not necessarily result in a reduction in the significant contribution spousal caregivers make to community dementia care, since the vast majority of participants also reported providing this care voluntarily. Indeed, on a 9-point scale from “no choice” to “completely voluntary,” the average response was well above the mid-point, with a median and a mode of 9. This seeming contradiction in our data perhaps highlights the distinction between choice with respect to becoming a caregiver and willingness to provide care. Indeed Arksey and Glendinning (2007) highlight the importance of differentiating between choice in taking on a caregiving role in the first place and choice within the caregiving role. Free choice in taking on a caregiving role is constrained by intrinsic and external factors, including a sense of duty to provide care and the lack of viable alternative options. Thus, arguably, a dichotomous yes/no question on caregiver choice is perhaps of limited value since few people will have a black and white situation in terms of choice to become a caregiver. Nevertheless, despite not freely selecting themselves into this role, most caregivers want to provide such care. Giving caregivers – as well as people living with dementia – more choice and say in how and where care is delivered is therefore an important objective that will help to ensure the acceptability and appropriateness of care for a given individual rather than necessarily leading to a reduction in the involvement of unpaid caregivers. Indeed, our findings suggest that enhancing caregivers’ perceptions of choice may actually help to delay placement in long-term care.

Although the lack of choice in taking up the caregiving role was not associated with placement in long-term care at follow-up in our sample, caregivers who perceived a lower degree of choice in respect to their caregiving at baseline were less likely to still be caring at home 1 year later. While this may seem like a contradiction, that caregivers cease a role they feel they have no choice in carrying out, it is clear that caregiving typically does not end when the care-recipient moves into long-term care, and the decision to institutionalize is often made at crisis points, such as during hospitalization, and is contingent on many extraneous factors including the availability of long-term beds and the financial means to avail of acceptable alternative care options (Robinson-Whelen and Kiecolt-Glaser, 1997; Arksey and Glendinning, 2007). Our findings suggest that caregivers who perceive a greater degree of choice in providing care may be more likely to decide against long-term care in such circumstances. This suggests that enhancing caregivers’ perceptions of choice in caregiving may actually help to prolong care in the community.

Nevertheless, while the degree of choice was a significant predictor, it explained little of the variance in the model. This may be in part because less than 20% of the sample had moved into long-term care at follow-up; further research with a larger sample and a longer follow-up time is needed to examine whether caregiver choice decreases the likelihood of institutionalization and the mechanisms through which this may take place. For example, lack of choice may impact negatively on the quality of care for the care-recipient as caregiver burden and distress is associated with worsening BPSDs and a greater likelihood of mistreatment and abusive behaviors toward the care-recipient (Orfila et al., 2018; Stall et al., 2019). Alternatively, lack of choice may lead to premature cessation of care by impacting on caregivers’ own health and wellbeing. However, while a positive relationship between choice and caregiver wellbeing has been reported by previous studies (Robinson-Whelen and Kiecolt-Glaser, 1997; Winter et al., 2010; Schulz et al., 2012; Al-Janabi et al., 2018), this was not supported by our data.

The finding that lack of choice, both in terms of taking up the caregiving role and the perceived degree of choice in caregiving, was not associated with poorer caregiver health, and well-being outcomes was unexpected. It is, however, in line with one study which found that willingness to care did not significantly predict quality of life or strain (Rand et al., 2019). It is possible that our findings differed from other studies because we assessed relationships over time, rather than cross-sectionally; however, few significant correlations were observed between our measures of choice and caregiver outcomes even at baseline. Indeed, the only outcome – aside from placement in long-term care – that was predicted by choice was the perception of positive aspects of care in that caregivers who reported that their care was more voluntary at baseline were more likely to identify positive aspects of care at follow-up. It is possible that, regardless of the constraints involved in having a free choice to care, *perceiving* caregiving to be their choice may benefit caregivers by functioning as a coping strategy that gives them a greater sense of control and enables them to view caregiving as in line with their personal values rather than as a duty they are obliged to carry out (Al-Janabi et al., 2018). This may in turn facilitate caregivers in finding the positives in the situation. Indeed according to self-determination theory (Deci and Ryan, 2008), more autonomous pro-social acts that are volitional rather than motivated by intrinsic or extrinsic pressures are more likely to have beneficial effects with regard to psychological and physical health. Thus, perceiving a greater degree of choice in caregiving may facilitate role adjustment and acceptance, enabling caregivers to perceive the benefits, or positive aspects of caregiving.

### Implications

Future research should be especially mindful of the way in which choice is assessed; our findings highlight that a dichotomous item is likely to give a different impression of the prevalence of choice and how choice relates to caregiver outcomes from a multiple option degree-of-choice measure. Since few people are likely to have an entirely unconstrained choice regarding whether or not to provide care that can be captured using a yes/no item, a more nuanced exploration of motivations to care, and the organizational and contextual factors that constrain choice would be of greater value.

Although our findings do not support the idea that limiting caregiver choice is related to poorer outcomes, this does not take away from the importance of choice in and of itself. It is a sad reflection of the social care system that the vast majority of caregivers did not feel they had a choice in taking up the responsibility of care and this points to the need for health and social care providers to be more mindful of caregivers’ perceptions of choice and how they might help to facilitate greater autonomy. For example, the availability and accessibility of current and accurate information on available service options is a prerequisite to exercising choice in relation to caregiving (Arksey and Glendinning, 2007). Nevertheless, the difficulty of accessing information about service use has consistently been reported (Department of Health, 2014a). Health and social care professionals should therefore ensure that potential caregivers are informed of all of the available options for care as well as the support services that are available to them if they choose to provide care at home.

While a stated objective of the Irish National Dementia Strategy (Department of Health, 2014a) is to support and facilitate people with dementia to live at home, in reality, insufficient services and supports exist in the community. Caregiver choice is thus constrained by many external organizational factors, including the limited availability and restricted range of care services, overstretched budgets for statutory services and restrictive eligibility criteria (Arksey and Glendinning, 2007). In this context, it may not seem feasible or economically viable to promote greater caregiver choice by making more alternative funded care options available to caregivers. Nevertheless, our findings indicate that, while social care systems rely on caregivers to provide care at home, enabling caregivers to have a greater degree of choice may not necessarily result in fewer caregivers choosing to do so. Rather, increasing perceptions of choice may actually help to delay placement in long-term care. Greater perceptions of choice can be fostered through the personalization of care options and allowing caregivers to self-direct the support services they access (Larkin and Mitchell, 2016). For example, by giving potential caregivers cash payments or personal budgets for services to organize their own care arrangements instead of allocating services (Arksey and Glendinning, 2007; Larkin and Mitchell, 2016). Greater perceived choice could also be facilitated by helping caregivers to explore additional sources of support for care in their communities, encouraging and assisting caregivers to distribute care tasks among other family members, or through interventions aimed increasing caregivers’ perceived control and confidence around care. Finally, caregiver choice can be promoted through policy measures by adopting a co-client approach, in which caregivers’ own interests and wellbeing are considered as key outcomes in their own right (Arksey and Glendinning, 2007). Considering the interests of caregivers and care-recipients together, will help to ensure that both have the ability to make meaningful choices.

Even when alternative options for care are available, caregivers may constrain their own choices because of feelings of obligation or duty to the care-recipient or by imposing limits on what they consider appropriate or necessary to get help with. For example, some caregivers may not consider it acceptable for someone else to carry out personal care tasks, while others may not be comfortable getting support with more general household chores (Arksey and Glendinning, 2007). Furthermore, it may not always be possible to balance the choices of care-recipients and caregivers. If care at home is the care-recipient’s preference and there is nobody else who could provide such care at home, then a potential caregiver’s “choice” is constrained by the absence of other meaningful options; choosing between providing care and not providing it is not a free choice. Thus, it is arguably unfeasible to provide potential caregivers with the opportunity to make completely free choices around care. However, at the very least, caregivers should be facilitated in having meaningful discussions about the options that exist and how these could best meet their own and the care-recipients’ needs.

### Strengths and Limitations

Our data are based on a relatively large sample of dementia caregivers and present the first prevalence data regarding spousal dementia caregiver choice. Furthermore, the inclusion of longitudinal data, and the examination of relationships between caregiver choice and caregiver outcomes over time is another strength of this study. Nevertheless, our findings are limited by the lack of data on the context in which caregivers took on their caregiving role or the actual alternative options that were available to them. We measured choice in an oversimplified manner using only a dichotomous item and a Likert scale. Therefore we have no information on participants’ positive motivations for caregiving or the reasons for their perceived lack of choice. Our findings are also limited by the homogenous nature of our sample, which included only spousal caregivers. The experiences of adult children or other relatives or friends caring for someone with dementia are likely to be very different, particularly if they perceive a lack of choice in adopting a caregiving role. In addition, while placement in long-term care and the identification of positive aspects of care at follow-up were predicted by degree of choice at baseline in our data, these findings should be interpreted with caution given the number of outcomes measures we examined and, therefore, the greater possibility that these findings emerged based on chance alone.

## Conclusion

For the vast majority of spousal dementia caregivers in Ireland, taking up the caregiving role is not perceived as a choice; yet, most caregivers report performing this role voluntarily, suggesting that facilitating greater choice would not necessarily diminish the key contribution family caregivers currently make to the dementia care system. Although, we found no evidence that a greater degree of choice predicted more positive health and wellbeing outcomes for caregivers over time, the perception of choice for caregivers is important in and of itself, and this should be reflected in dementia care policy. Health and social care professionals should give due consideration to caregivers’ perception of choice and facilitate caregiver preferences wherever possible by carefully outlining and exploring the care options available and by working with caregivers to increase their confidence in providing care. Providing caregivers with greater choice with regards to care may benefit caregivers by facilitating the identification of positive aspects of care and could potentially delay care-recipient placement in long-term care.

## Data Availability

The datasets for this study will not be made publicly available because the participants did not give consent for their data to be shared outside of the research team.

## Ethics Statement

This study was carried out in accordance with the recommendations of the Trinity College Dublin School of Psychology Ethics Committee with written informed consent from all participants. All participants gave written informed consent in accordance with the Declaration of Helsinki. The protocol was approved by the Trinity College Dublin School of Psychology Ethics Committee.

## Author Contributions

MP, SB, IR, and BAL conceived and designed the study. MP performed the analysis. MP and AS-R wrote the first draft of the manuscript. All authors revised the manuscript and approved it for submission.

## Conflict of Interest Statement

The authors declare that the research was conducted in the absence of any commercial or financial relationships that could be construed as a potential conflict of interest.
